# A Method of Codec Comparison and Selection for Good Quality Video Transmission Over Limited-Bandwidth Networks

**DOI:** 10.3390/s21134589

**Published:** 2021-07-04

**Authors:** Janusz Klink

**Affiliations:** Department of Telecommunications and Teleinformatics, Wroclaw University of Science and Technology, 50-370 Wroclaw, Poland; janusz.klink@pwr.edu.pl

**Keywords:** video quality assessment, dynamic adaptive streaming, MPEG DASH, coding efficiency, codec comparison, spline interpolation, H.264 (AVC), H.265 (HEVC), AV1

## Abstract

Finding a proper balance between video quality and the required bandwidth is an important issue, especially in networks of limited capacity. The problem of comparing the efficiency of video codecs and choosing the most suitable one in a specific situation has become very important. This paper proposes a method of comparing video codecs while also taking into account objective quality assessment metrics. The author shows the process of preparing video footage, assessing its quality, determining the rate–distortion curves, and calculating the bitrate saving for pairs of examined codecs. Thanks to the use of the spline interpolation method, the obtained results are better than those previously presented in the literature, and more resistant to the quality metric used.

## 1. Introduction

A huge growth in Internet traffic has been observed in recent years. According to the Cisco’s Visual Networking Index (VNI) forecast, by the year 2022, more Internet protocol (IP) traffic will cross global networks than all the traffic observed before 2017. Busy-hour Internet traffic (the busiest 60 min period in a day) has grown even more (i.e., by a factor of 4.6) than average Internet traffic (by a factor of 3.2) in the years 2016–2021. Moreover, video will account for up to 82 percent of global Internet traffic in 2022 [1,2]. In this context, proper resource management in the case of low-bandwidth networks, or their segments, plays a crucial role. The delivery of good quality video content may be a challenging task owing to the limitations of the last mile-, wireless-, or sensor networks. In the case of sensor networks, their resource constraints, especially in terms of processing capability, memory, battery, and achievable data rates, may seriously decrease the offered quality of service (QoS) [3]. Thus, the implementation of proper multimedia source coding techniques, in order to achieve less demanding video content, may help to solve this problem. The main objectives of designing a coder for sensor networks are high compression efficiency and low complexity in order to limit bandwidth and energy consumption. A further challenge is the provision of the robust and error-resilient coding of source video. However, the delivery of good quality video is not only an important issue in the case of specific environments like sensor networks, and this problem can be discussed in a much wider context.

The Internet is currently the most popular and broadly available means of communication, and is it used for video streaming to almost every place in the world. Data streaming must be adapted to the dynamically varying circumstances, while also taking into account different network parameters. This idea was implemented in the Internet as adaptive streaming over the hypertext transport protocol (HAS). It was initially introduced by leading companies like Apple (HTTP live streaming) [4], Adobe (HTTP dynamic streaming) [5], and Microsoft (smooth streaming) [6]. Subsequently, ISO/IEC (2014) proposed an open and vendor independent standard that describes dynamic adaptive streaming over HTTP (DASH), which was then ratified by the Moving Pictures Experts Group (MPEG-DASH) [7,8,9,10].

**Figure 1:**
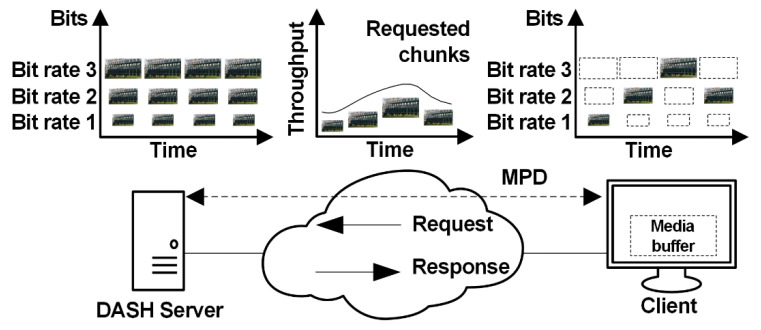
Communication in HTTP adaptive streaming systems. MPD, media presentation description; DASH, dynamic adaptive streaming over HTTP.

In the case of the above mentioned techniques, clients pull video from a standard HTTP server, which hosts the media content, using HTTP as the application, and the transmission control protocol (TCP) as the transport-layer protocols. The video is stored on the HTTP server as a stream of media segments (chunks) that are typically two to ten seconds long. The segments are encoded at multiple bitrate levels and listed in a media presentation description (MPD) file that provides an index for the available segments at the server. The adaptation streaming mechanism assumes that a DASH client estimates the available network bandwidth and uses information from the playback buffer to select a suitable bitrate of the video chunk to be requested (using GET message) from the server. This process, called bitrate switching, allows sufficient data to be kept in the playback buffer in order to avoid video stalls and to preserve a seamless, or at least smoother, streaming experience [11]. When the network bandwidth is severely limited, lower resolutions and coding rates are used, and when the situation improves, it reverts to higher resolutions and coding rates [12,13] (see Figure 1).

The dataset should be chosen so that efficient and unobtrusive switching between different video resolutions is possible. This is required in order to preserve a high, and possibly stable, video quality. The quality may be defined by objective parameters that describe quality of service (QoS) [14,15,16,17,18], or by the users’ subjective assessment scores that represent the so-called quality of experience (QoE) [19,20,21]. In this paper, the objective approach will be presented as a method of assessing video quality and comparing codec performance. A set of objective methods may be divided into full-reference (FR), reduced-reference (RR), and no-reference (NR) methods, respectively. FR methods assume that there is access to both the video footage (reference) and the distorted (tested) video samples, which are subsequently compared with the reference [22]. When only partial information regarding the source video is available, RR methods are used [23]. In the case of NR methods, there is only access to the distorted signal, and video quality estimation is performed without any knowledge of the source video footage [24]. In order to examine two codecs, it is worth comparing not one, but several video samples that are encoded using these codecs. The preparation of a proper video dataset representation is a very important and time-consuming task [25,26]. It should take into account different parameters, e.g., different spatial and temporal resolutions of the video, coding bitrates, color bit depth, and chroma subsampling schemes. This process should be performed individually for each prospective video codec.

Currently, H.264/AVC (audio video coding) [27] and H.265/HEVC (high efficiency video coding) [28] are the two ITU-T (International Telecommunication Union—Telecommunication Standardization Sector) coding standards that are commonly used by video content delivery platforms. In 2018, a new video coding standard called AV1 [29] was proposed by the Alliance for Open Media, which gathers the leaders and innovators of the IT industry, as well as cutting-edge streaming platforms or broadcasting companies like IBM, Cisco, Microsoft, Apple, Facebook, Netflix, Vimeo, Hulu, and so on.

A comparison of two codecs may be performed by calculating the difference between the obtained video quality levels for a specific coding bitrate. The second approach assumes the opposite situation, i.e., determining the coding bitrates needed to achieve a required quality level. The calculated difference between these coding bitrates is called a delta bitrate (D-BR), which denotes the bitrate saving that can be achieved using one codec instead of the other one. Such a method of measuring the coding efficiency for the examined codec in comparison with the other one was proposed by Gisle Bjöntegaard in [30], where the relations between the video quality offered by the two codecs as a function of the coding bitrate are represented by rate–distortion (R–D) curves.

The Bjöntegaard model is a very, if not the most, common method used to compare video codecs performances, where the quality is represented by the PSNR metric. Thus, the results of comparing codecs’ performances are expressed by Bjöntegaard delta PSNR (BD-PSNR) or Bjöntegaard delta bit rate (BD-BR) values. It is known from the literature that PSNR is not always the best factor describing video quality, especially in comparison with subjectively measured quality—the quality perceived by the users. Objective quality metrics, especially PSNR, may give different results depending on the specific video content [31,32]. Laude et al. indicate the main reasons for the difficulty of comparing video codecs, where they underline the very important role played by codec implementations, which are often different. Although the coding standards are precisely described, they cannot be directly evaluated by simulations or laboratory tests. The quality assessment concerns specific implementations of the standards. Sometimes, two encoder implementations based on the same standard or recommendation can differ [33]. The second factor that plays an important role is codec configuration. Encoders can be configured in many different ways, e.g., taking into account different rate–distortion optimization settings [34]. Some codec implementations allow presets that define different trade-offs between computational complexity and their coding efficiency. Depending on these settings, the bit rates can differ significantly for a video encoded at the same quality [33]. Next, usage of the intra coding and/or motion compensation plays a role in coding efficiency Although intra coding plays a huge role in video coding applications, videos without motion-compensated pictures can require even 100 times the bit rate of motion-compensated footages to achieve the same quality [35]. Moreover, the group of pictures (GoP) is an important issue that can influence the results of codecs’ comparison. Depending on the available reference pictures, the efficiency of motion-compensated prediction can be different [36]. Finally, the metrics used for the quality assessment matter. PSNR is often used because it is simple to calculate and has clear physical meanings. It also presents relatively good results when assessing the influence of degradation factors on a specific video clip. In such a situation, we compare the quality of the same video before and after the degradation process. The results correlate with the quality perceived by the users taking part in the subjective assessment procedure [32]. On the other hand, it can be noted that PSNR values may significantly differ when comparing completely different video samples even if the examined source material is not distorted. It confirms that the content matters here [33]. New Quality Index (NQI) is a metric that was established in order to define a universal quality measure that may be used to model the distortion of the image [17]. Although NQI is a more complicated metric than PSNR, it does not solve all the problems with the quality assessment of different video materials (cf. results presented in [32]). In such situations, PSNR may gain an advantage, especially when it is more popular and accessible in different applications. However, PSNR is memoryless, which means that it is calculated pixel by pixel, independently, for each pair of corresponding frames of the two compared videos. It does not take any spatial and temporal relationships between pixels of the video footage. When the reference and examined videos are randomly reordered in the same way, the PSNR between them will stay unchanged. However, textures, patterns, and so on matter in this case. Moreover, ordering of the samples carries important perceptual structural information about the contents of the visual scene [37]. Thus, it is worth considering other video quality metrics like the structural similarity (SSIM) index [38,39], which takes into account the fact that natural image signals are highly structured. The Bjöntegaard model might not be an accurate predictor of the true coding efficiency as it relies on PSNR measurements where the average bit rate difference for the same quality between four data points is calculated [33,40]. Moreover, it may cause problems with analyzing high resolution images, where higher ranges of coding bitrates are taken into account. Then, building models based on more than four data points seems to be reasonable. In consequence, this model based on higher order polynomials will be more susceptible to Runge’s phenomenon, which additionally may result in inaccurate BD evaluations [41,42]. In order to overcome this problem, the author uses, in this paper, a spline interpolation as the method of fitting the R–D curves. A more detailed description of spline interpolation method and its implementation can be found in the literature [43].

The main aspects of the paper are as follows:

(a) Presentation of a new approach to comparing the performance of video codecs;
(b) Showing the whole video quality assessment process—the preparation of video footage and test material, the assessment of the quality of individual samples, and the presentation of results;
(c) Implementation of the spline interpolation method for building R–D curves for the examined codecs;
(d) Presentation of the results of comparing the H.264, H.265, and AV1 codecs, which are more quality-metric resistant than those previously presented in the literature.

The paper is organized as follows. The next section presents the materials and methods used to compare video codecs, with the preparation process of video footages being shown step-by-step. Next, the methods of comparing codecs, based on a limited set of video samples, are discussed and the way of solving the problem is proposed. Validation of this approach is then conducted. The ‘Results’ section presents the main outcome of the author’s research on comparing codecs, and includes some experimental conclusions. Finally, an interpretation of the results is conducted and future research directions are highlighted.

## 2. Materials and Methods

One issue that complicates the comparison process of two video codecs is that the relation between the curves, which present video quality as a function of the coding bitrate, is not a constant value for the two specific codecs. Thus, the coding efficiency of one codec in comparison with the other one may be represented as a function of the area between the R–D curves [44]. Each curve represents a relationship between the coding bitrates and the achieved video quality values. Thus, the (delta) distortion may be presented as follows:

ΔD = E[D_1_ − D_2_]. (1)

**Figure 2:**
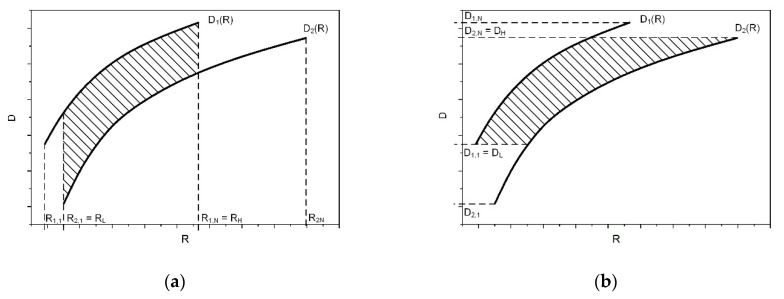
Calculation of the delta distortion (**a**) and delta rate (**b**).

As presented in Figure 2a, a rate–distortion curve for each of the two examined codecs is given by a set of *N* bitrates (*R_X,1_*, …, *R_X,NX_*, where *X*-index denotes the curve number), with the corresponding video quality being represented by the appropriate PSNR or other measured quality values. In general, these measurement results are denoted as *D_X,1_*, …, *D_X,NX_*. The results may also show the quality distortion as a function of the coding bitrate. A functional relation between the coding bitrates and the corresponding quality values may be described by third-order logarithmic polynomial fitting, as presented in Equation (2):

D_F_(R) = a ∗ log^3^R + b ∗ log^2^R + c ∗ logR + d (2)

where D_F_ is the fitted distortion (in Bjöntegaard model based on PSNR); R is the coding bitrate; and a, b, c, and d are the parameters.

In order to validate the results based on the PSNR values, other objective quality measures, such as SSIM [45], may also be used. In the second case, the Bjöntegaard delta SSIM (BD-SSIM) is the product of these calculations, but the results cannot be directly compared because both the PSNR and SSIM metrics use different scales, i.e., PSNR values are expressed in decibels, while SSIM is represented by an absolute value in the range from 0 to 1. A solution is to calculate the Bjöntegaard delta bitrate (BD-BR), which is defined as the average value of subtraction of the coding bitrates corresponding to a given set of video quality levels for the examined codecs. This is presented by Equation (3):

ΔR = E[(R_2_ − R_1_)/R_1_] = E[R_2_/R_1_] − 1 = E[10^(r_2_ − r_1_)] − 1 ≈ 10^E[r_2_ − r_1_] − 1. (3)

Assuming that the logarithm of the coding rate may be expressed as a function of the distortion by a third–order polynomial like the following:

R_F_(d) = a ∗ D^3^ + b ∗ D^2^ + c ∗ D + D, (4)

the average delta bitrate (see equation 3) may be presented as follows:

ΔR ≈ 10^[(1/(D_H_ − D_L_)) ∗ INT(r_F1_(D) − r_F2_(D))dD|D_L_ to D_H_)] − 1 (5)

where D_L_ and D_H_ are the lower and higher integration limits, respectively, which may be designated as follows:

D_L_ = max{min(D_1,1_, …, D_1,N1_), min(D_2,1_, …, D_2,N2_)}; D_H_ = min{max(D_1,1_, …, D_1,N1_), max(D_2,1_, …, D_2,N2_)}. (6)

A graphical explanation of this approach is presented in Figure 2b.

The performance of the previously mentioned video codecs will be evaluated using an objective quality assessment method. In this paper, the FR method will be used in order to determine the video quality of the three examined codecs, i.e., H.264, H.265, and AV1, as a function of the coding bitrate. In the first step, the source video footage and a set of test samples of the same time and spatial resolutions (of different bitrates) should be prepared. It should be noted that, in order to have a reference sample of the highest quality, the source video must be lossless (i.e., uncompressed) footage. This often requires the use of a professional camera in order to record the video; however, most consumer class devices, including ubiquitous smartphones, usually save the captured video using a lossy compression. As a consequence, the evaluation of the performance of the video codecs presented in the paper was carried out using two kinds of video footage taken from an open database, which is provided on the website http://ultravideo.cs.tut.fi/#testsequences (accessed on 3 July 2021) by the Ultra Video Group (UVG) [46]. The first video, originally called ‘Beauty’, presents a lady’s head on a black background, with her hair fluttering in the wind. There are relatively few details and little movement in this video clip. The second footage, called ‘ReadyStGo’, presents the start and the first few seconds of a horse race. This video presents a lot of details and there is a lot of movement in it. Both footages were originally captured using a Sony F65 camera as an uncompressed video and were provided on the UVG web page, as RAW (.yuv) videos, in two spatial resolutions, i.e., 3840 × 2160 pixels (4 K) and 1920 × 1080 pixels (1080 p). Based on 1080 p video, new video footage of smaller spatial resolutions, as presented in the author’s earlier study [32], was prepared. The reference footage taken into account in this paper is uncompressed and 24 s long, with an 8-bit color depth and 4:2:0 chroma subsampling at the resolution of 858 × 480 pixels (480 p). It contains 25 frames per second (fps) with an overall bitrate of 124 Mbps. The author chose this resolution because it is one of the resolutions (in the middle of 240 p and 1080 p) recommended by streaming platforms, like YouTube, for providing video streams in the IP network using the MPEG-DASH technique. From the perspective of the research and presented methods and results, the chosen spatial resolution of the examined video footages is of less importance. However, the main reason for choosing this resolution was the reasonable processing time of such video samples during the assessment process, as well as the relatively low size of the files when saved on the hard disk.

In order to prepare the reference video files and a series of test samples, the ffmpeg framework [47] was used. Rescaling of the original 1080 p video footage into the 480 p format was done using the following command:


ffmpeg -s:v **1920x1080** -i **input.yuv** -vf **scale=858:480** -c:v rawvideo 
-pix_fmt yuv420p **output.yuv,**
	  

where input.yuv and output.yuv are the analyzed video footages, i.e., ‘Beauty’ and ‘ReadyStGo’, before (beauty_raw1080p.yuv and readystgo_raw1080p.yuv) and after (beauty_raw480p.yuv and readystgo_raw480p.yuv) the rescaling operation, respectively. The two video footages of the lower resolution were used to prepare the test video files; three analyzed codecs, i.e., H.264, H.265, and AV1, and different coding bitrates, were taken into account.

ITU-T recommendations on the subjective video quality assessment methods propose the use of video samples with a length of no more than 10 s in order to reduce the test time. This takes into account that one test sequence may contain several video samples that should be assessed. However, it should be noted that such video footage may sometimes be too short in the case of using subjective methods, when it is actually humans that should assess the video quality. Therefore, the recommendation says that the presentation time (equal to the video sample’s length) may be increased according to the content of the test material [48]. Based on YouTube recommendations for 480 p video, the following coding bitrates were used for the video test samples: 500 kbps, 600 kbps, 700 kbps, 800 kbps, 900 kbps, 1 Mbps, 1.5 Mbps, and 2 Mbps. The author’s earlier study [32] confirmed that this range of bitrates guarantees an acceptable quality of the examined videos for the mentioned spatial resolution.

The video test samples were prepared and saved as mp4 files using the following ffmpeg commands:

(a) for the H.264 encoded samples


ffmpeg -f rawvideo -pix_fmt yuv420p -s:v 858x480 -r 25 -i
***ref_file.yuv*** –b:v ***bitrate_in_bps*** -c:v libx264
***test_480p_h264_N_file.mp4***

(b) for the H.265 encoded samples


ffmpeg -f rawvideo -pix_fmt yuv420p -s:v 858x480 -r 25 -i
***ref_file.yuv*** –b:v ***bitrate_in_bps*** -c:v libx265
***test_480p_h265_N_file.mp4***

(c) for the AV1 encoded samples


ffmpeg -f rawvideo -pix_fmt yuv420p -s:v 858x480 -r 25 -i
***ref_file.yuv*** –b:v ***bitrate_in_bps*** -c:v libaom-av1 –strict -2
***test_480p_av1_N_file.mp4***
			

where, as a *ref_file.yuv,* the previously prepared reference video samples, i.e., beauty_raw480p.yuv and readystgo_raw480p.yuv, were taken. The test files named as *test_480p_h264_N_file.mp4, test_480p_h264_N_file.mp4, *and* test_480p_h264_N_file.mp4 *were H.264-, H.265-, and AV1-encoded video samples, respectively. After these operations, six sets of video test samples were obtained—taking into account three codecs multiplied by two different reference files of eight coding bitrates. This makes 48 video test samples in total (Table 1).

In the next step, the quality of each video sample was determined by calculating the appropriate PSNR and SSIM values using a video quality estimator (VQE) tool [49]. Based on these objectively determined quality values, a comparison of the examined video codecs could have been done. In order to calculate the delta bitrate (D-BR), the differences between the coding bitrates corresponding to the same video quality levels, for each codec, must be known. The issue is that it is very difficult to define the quality of the encoded video sample explicitly during the encoding process using an ffmpeg tool. The quality is a non-linear function of the defined coding bitrate. Moreover, declaring a specific target bitrate of the test video sample, as an ffmpeg parameter of the encoding process, does not result in obtaining a video sample of exactly the same bitrate as previously specified.

Usually, the obtained video encoding bitrates are close to the specified ones, but not exactly the same (see Tables 3 and 4). Moreover, taking into account the limited number of compared video samples of different bitrates, the quality values of the samples presented by the first codec are usually not the same as the quality of the samples generated by the second codec. This is a consequence of the video sample preparation process based on ffmpeg. A real encoding bitrate of a video sample, obtained during ffmpeg conversion from a raw video, usually is close, but not equal, to the specified ‘target bitrate’. On the other hand, analysis of bitrates of video samples presenting the same quality level is also difficult because the obtained sets of footage are of little different bitrates and qualities.

**Figure 3:**
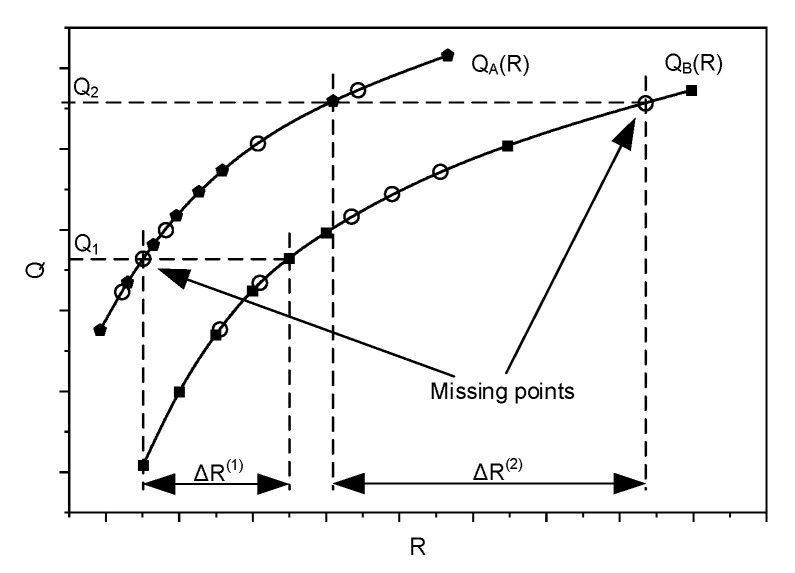
Calculation (issue) of the delta rate based on the real video samples.

As presented in Figure 3, in the two compared sets of video samples, there are usually no samples representing the same quality. If, in the first group of video samples, there is one of a given quality (represented by the appropriate PSNR or SSIM value), then there is usually a lack of the same quality video sample in the second group, meaning that there are missing points representing the same quality levels for the compared codecs (see Figure 3).

**Figure 4:**
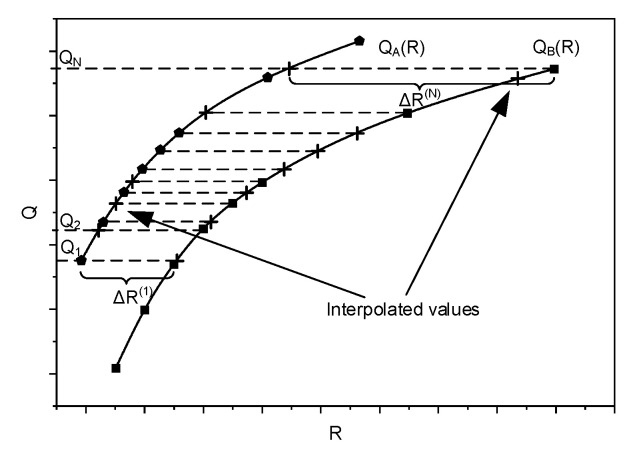
Calculation of the delta rate between the two codecs—based on real video samples and interpolated values.

The appropriate curve fitting to the points presented in Figure 3, and the building of a mathematical model that allows the missing quality values to be found, may lead to the solution of this problem. A proper interpolation method should be used to fit the curve to the points obtained as the result of the quality measurements for the selected coding bitrates. BD metrics [40,50] are very useful for comparing pairs of codecs; however, they can give unexpected results in the case of ultra-high-definition (UHD) video sequences [51]. In order to compare two codecs in a wider range of quality or bitrate values, more measurement points (more prepared video samples) would be needed, which could be a very time consuming process. Determining the missing values by interpolation may help in solving the problem. As mentioned before, polynomial interpolation, implemented by the Bjöntegaard model, is susceptible to Runge’s phenomenon and may result in inaccurate BD evaluations. Implementing a spline interpolation, as the method of fitting the R–D curves, allows the author a piecewise (here, third-order) polynomial to be used instead of fitting a single third- or higher-order polynomial, as well as comparable, or even better results, to be achieved. When the fitting (R–D) curves are determined, the bitrate saving for each pair of codecs can be calculated for a given range of quality values. This, as mentioned before, may be determined using the Bjöntegaard model, or it may also be done by calculating the area between two R–D curves and dividing the result by the given (quality) distortion range. The author used both approaches, and the pair of them gave comparable results. Nevertheless, in both cases, the results are burdened with errors resulting from the numerical integration of the R–D curves. On the other hand, the sets of bitrates in the assumed range, as well as the corresponding quality values, are countable and limited. Thus, the comparison of the codecs may be done much more easily: the author’s approach assumes that the quality values are determined, based on the fitted R–D curves, for each single bitrate in the assumed range. Therefore, based on spline interpolation, the quality (PSNR and SSIM) values for each bitrate were calculated. In the next step, the differences between the bitrates corresponding to the same quality levels of the compared codecs were calculated (Figure 4).

**Figure 5:**
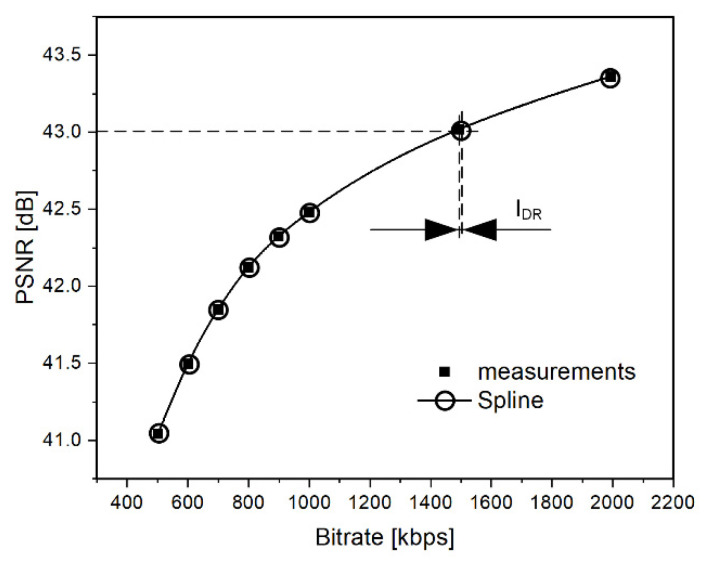
Calculation of the ‘interpolation delta rates’.

Finally, for each pair of codecs, the average value of bitrate distortion was calculated (see Tables 5 and 6). These calculations were done for two sets of video footage and two quality metrics. The interpolation uncertainty may lead to inequalities between the calculated and the real (measured) coding bitrate values corresponding to the appropriate quality levels for the analyzed video footages. Therefore, the author’s approach was validated by calculating the ‘interpolation delta rates’ (IDR), i.e., the differences between the values of the encoding bitrates of (a subset of) the real video footage and the corresponding values of the bitrates of (a set of) the points obtained from spline interpolation, which took into account appropriate quality (PSNR and SSIM) levels (see Figure 5).

Table 2 presents the measured and the interpolated values of the coding bitrates for a chosen set of video quality values for the beauty_raw480p.yuv H.264-encoded video footage. The IDR values varied depending on the metric and codec that were used. In the case of the H.265 codec, the IDR values were IDR_PSNR_ = 0.31% and IDR_SSIM_ = 0.34%. Such low IDR values (less than 1%) show that the interpolated PSNR and SSIM values allow good R–D (rate–distortion) curves to be built for the H.264 and H.265 codecs. The research on the AV1 codec showed better interpolation results for the SSIM metric than for the PSNR metric. In this case, the obtained IDR values were IDR_PSNR_ = 12.53% and IDR_SSIM_ = 4.94%.

The next section presents the interpolation R–D curves determined for the two sets of video footage, i.e., the ‘Beauty’ and ‘ReadyStGo’ footage, encoded using the H.264, H.265, and AV1 codecs. Next, based on these curves, the results of comparing these codecs are discussed.

## 3. Results

As already mentioned, the video quality assessment was conducted based on a limited number of prepared video footages, i.e., 8 samples per each examined codec (24 samples in total). For each encoded video sample, the values of PSNR and SSIM were determined.

### 3.1. Results of the Objective Quality Assessment for the Examined Video Samples Using Different Codecs

Table 3 and Table 4 present the PSNR and SSIM values measured for the ‘Beauty’ and ‘ReadtStGo’ video samples, respectively.

Each table contains the PSNR and SSIM results obtained using video quality estimator—a software tool for video quality assessment provided by Elecard Company [49]. The ‘Target bitrate’ column contains the bitrate values that were set up, as an ffmpeg parameter, during the encoding process. The real bitrates achieved for each codec are presented in the appropriate ‘Bitrate’ columns. A rough comparison shows that the H.264-encoded videos had the lowest quality, while the AV1 codec allowed the best quality to be achieved.

A more detailed analysis could be carried out after the interpolation of the measured PSNR and SSIM values, and after drawing the appropriate R–D curves for each codec and video sample.

### 3.2. Comparison of the R–D Curves for the Examined Codecs and Video Samples

**Figure 6:**
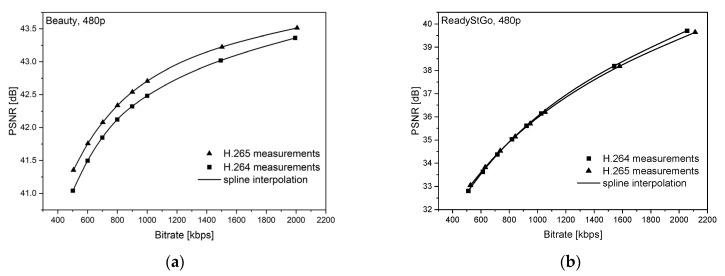
Comparison of the H.264 and H.265 codecs, based on the PSNR metric, for two different videos: (**a**) slow motion video; (**b**) fast motion video.

**Figure 7:**
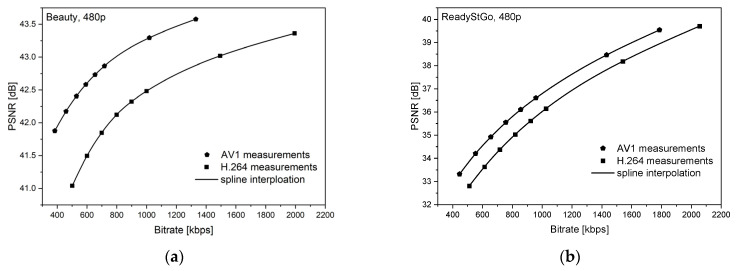
Comparison of the H.264 and AV1 codecs, based on the PSNR metric, for two different videos: (**a**) slow motion video; (**b**) fast motion video.

**Figure 8:**
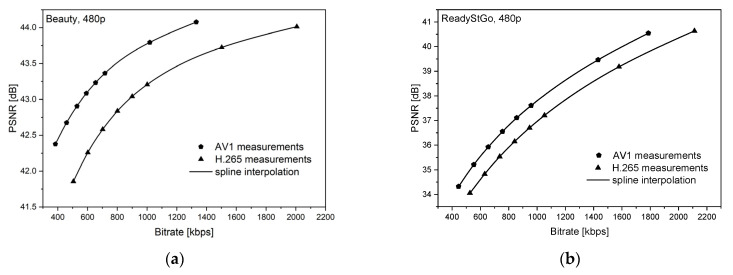
Comparison of the H.265 and AV1 codecs, based on the PSNR metric, for two different videos: (**a**) slow motion video; (**b**) fast motion video.

**Figure 9:**
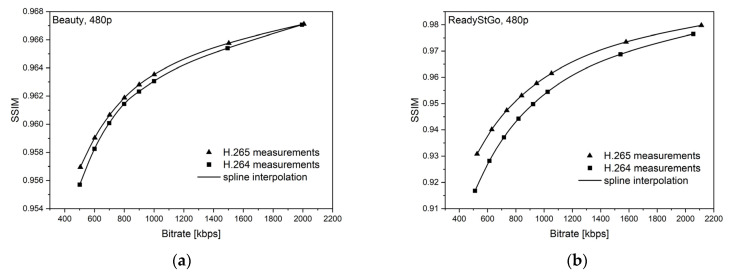
Comparison of the H.264 and H.265 codecs, based on the SSIM metric, for two different videos: (**a**) slow motion video; (**b**) fast motion video.

**Figure 10:**
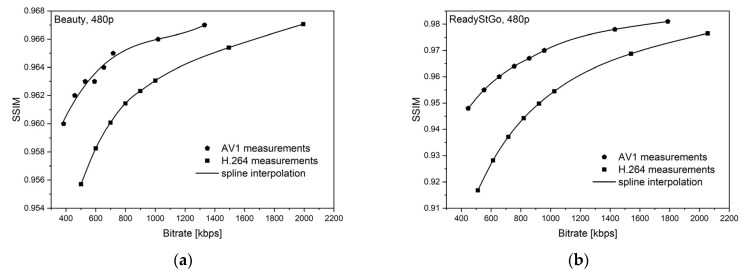
Comparison of the H.264 and AV1 codecs, based on the SSIM metric, for two different videos: (**a**) slow motion video; (**b**) fast motion video.

**Figure 11:**
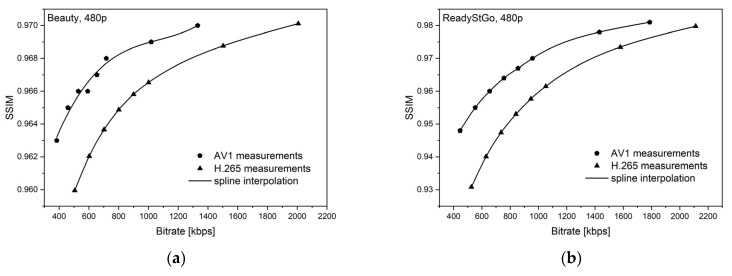
Comparison of the H.265 and AV1 codecs, based on the SSIM metric, for two different videos: (**a**) slow motion video; (**b**) fast motion video.

Figure 6, Figure 7, Figure 8, Figure 9, Figure 10 and Figure 11 present the results of comparing the H.264, H.265, and AV1 codecs, based on PSNR and SSIM metrics, for two different videos: (**a**) slow motion video (‘Beauty’); (**b**) fast motion video (‘ReadyStGo’).

From the curves presented in Figure 6, Figure 7, Figure 8, Figure 9, Figure 10 and Figure 11, some general conclusions can be drawn:

- Firstly, the observed video quality values, expressed by both the PSNR and SSIM metrics, are directly proportional to the coding bitrate. However, these relations are not linear;
- Secondly, the obtained results are consistent with those presented in the literature [33], where the AV1 codec presents the highest quality, with the H.264 codec achieving the lowest scores at the same reference bitrate;
- Thirdly, the R–D curves, describing a specific codec, differ from each other, depending on the metric and video footage used.

Thus, comparison of the codecs’ performances requires further discussion, as presented in the next section.

## 4. Discussion

Table 5 and Table 6 present the results of the codecs’ efficiency comparison performed by the author. The percentages of the bitrate savings were calculated, for each pair of codecs, based on the distortion–rate curves and the comparison of the coding bitrates that represent the corresponding video quality levels. The quality was expressed by the appropriate (measured and interpolated) PSNR and SSIM values that correspond to the coding bitrates in the range from 500 kbps to 2000 kbps. As mentioned before, this range of bitrates guarantees an acceptable quality of the examined footages for the 480 p videos [32], and at the same time generates a reasonably low data rate traffic during transmission over the network. Calculations of the average bitrate savings (ABS), expressed in ‘%’, were done using both the Bjöntegaard (BDR) and the author’s (DR) methods. Both the PSNR and SSIM metric showed the same winner in each pair of compared codecs, i.e., H.264 versus H265 (better), H.264 versus AV1 (better), and H.264 versus AV1 (better). However, the obtained bitrate saving results depended on the specific pair of codecs, metric, and even video sample used. In the case of the ‘Beauty’ video (see Table 5), the H.265 codec turned out to be more economical than the H.264 codec by more than 37% (i.e., 37.72% in the case of PSNR and 37.48% in the case of SSIM), while the AV1 codec was better than the H.265 and H.264 codecs by approximately 40% and 62%, respectively (depending on the metric used). The ABS values lower than zero in Table 5 and Table 6 denote the bitrate savings of the second codec (denoted by *) in relation to the first one.

According to Table 6, the H.265 codec saved c.a. 15% of the bitrate in comparison with the H.264 codec (i.e., 16.48% in the case of PSNR and 14.68% in the case of SSIM), while the AV1 codec was better than the H.264 codec by 48.26% (PSNR) and 44.79% (SSIM), depending on the metric used. The comparison of the H.265 and AV1 codecs also showed the advantage of AV1, i.e., 38.23% in the case of PSNR and 36.20% in the case of SSIM. It can be noted that the bitrate savings resulting from the application of different codecs strongly depend on the video footage, which may be of ‘slow’ or ‘high’ motion, or present less or more details, and so on. On the other hand, there are also differences between bitrate saving calculations based on different metrics, such as PSNR or SSIM. In general, lower differences are better, because such a bitrate saving, by definition, should not depend on the metric used; the metrics should be treated here as objective ‘measurement tools’ that show reasonable and comparable results.

In connection with this, the author’s calculation method seems to produce better results than the Bjöntegaard model [52], because the difference between results based on PSNR and SSIM are lower in the case of the author’s approach. The average **Δ_DR_** values are lower than the average **Δ_BDR_**, i.e., in the case of the ‘Beauty’ video, the avg. **Δ_DR_** = 0.81%, while the avg. **Δ_BDR_** = 1.98%, and in the case of the ‘ReadyStGo’ video, the avg. **Δ_DR_** = 2.43%, while the avg. **Δ_BDR_** = 2.63%. It can thus be noted that the author’s method of comparing codecs is more resistant to the metric used in the video quality assessment process.

The author’s method of comparing the performance of codecs, as well as the Bjöntegaard model, allow reasonable results to be achieved based on the interpolation of real video samples, even if their number is limited. Such a reduction in the number of the required samples allows the whole process to be accelerated, which is because the preparation of the test samples may take a long time [25,26]. This obviously depends on the coding parameters (like bitrate, number of frames per second, video resolution, and so on) and the complexity of the video scene. In the case of the analyzed 10 s long ‘Beauty’ and ReadyStGo’ video footage, the preparation time of one video sample, using a laptop with Intel Core i7 CPU/2.9GHz and 64-bit Windows OS, varied from several minutes to over 10 h. Therefore, most of the files (especially AV1-encoded videos) were processed in the supercomputing center of Wroclaw University of Science and Technology, where they were encoded using batch-mode processing.

The problem of choosing the right video coding method may also be discussed in much more detail. The author’s observations, as well as many reports in the literature [40,53], show that achieving a compromise between high compression and good video quality depends not only on the codec used, but also on the specific video footage to be processed and the encoding parameters used [31,33]. The interpolated R–D curves are highly dependent on the video content, e.g., motion and texture are very important aspects that influence video compression efficiency [44]. Therefore, big streaming platforms develop per-title encoding, where they run analyses on individual titles in order to determine the optimal encoding recipes based on their complexity (e.g., action scenes vs. unchanging landscapes or cartoons). On the other hand, when taking into account even a specific video footage and codec, there is a need to prepare the encoding ladder for dynamic adaptive streaming of the video to the user. The construction of such a ladder embodies the most significant decisions made by the professionals involved in the video delivery process, where a compromise between good quality experienced by the viewers and network and/or user application constraints must be ensured. In this sense, new methods and tools for an easier and reliable comparison of the quality of video footages and codec bitrate savings are very important. The examples of such efforts, based on rate distortion modeling, can be found in the literature, e.g., Battista et al. estimate R–D models for different sets of video footage and codecs using a piecewise cubic Hermite polynomials interpolation [54], and then they compute the average delta bitrate savings based on PSNR metric. The author of this paper goes a step further and uses cubic spline interpolation method in determining R–D curves on both PSNR and SSIM metrics. Such an approach allowed to achieve two goals, i.e., more precise R–D projection than proposed by the Bjöntegaard model and obtaining the results that are more similar to each other. Thus, the method presented here seems to be more resistant to the metric used.

## Figures and Tables

**Table 1:** The video test samples.

Reference Files: beauty_raw480p.yuv and readystgo_raw480p.yuv			
Target Bitrate ^1^ [kbps]	H.264 Encoded mp4 File	H.265 Encoded mp4 File	AV1 Encoded mp4 File
500	tvf_480p_h264_500k ^2^	tvf_480p_h265_500k	tvf_480p_av1_500k
600	tvf_480p_h264_600k	tvf_480p_h265_600k	tvf_480p_av1_600k
700	tvf_480p_h264_700k	tvf_480p_h265_700k	tvf_480p_av1_700k
800	tvf_480p_h264_800k	tvf_480p_h265_800k	tvf_480p_av1_800k
900	tvf_480p_h264_900k	tvf_480p_h265_900k	tvf_480p_av1_900k
1000	tvf_480p_h264_1000k	tvf_480p_h265_1000k	tvf_480p_av1_1000k
1500	tvf_480p_h264_1500k	tvf_480p_h265_1500k	tvf_480p_av1_1500k
2000	tvf_480p_h264_2000k	tvf_480p_h265_2000k	tvf_480p_av1_2000k

^1^ ‘Target bitrate’ means the coding bitrate as declared in ffmpeg during the encoding process. ^2^ The ‘tvf’ means the ‘tested video file’ and should be replaced by ‘beauty’ or ‘readystgo’, depending on the original reference video file used.

**Table 2:** Differences between the measured and interpolated coding bitrates for the H.264 codec.

Reference File: beauty_raw480p.yuv, Video codec: H.264							
		PSNR Metric			SSIM Metric		
Target Bitrate [kbps]	Measured Bitrate ^1^ (MB) [kbps]	PSNR [dB]	Interpolated Bitrate ^2^ (IB) [kbps]	IDR [%]	SSIM	Interpolated Bitrate (IB) [kbps]	IDR [%]
1100	1099	42.61	1095	0.36	0.9637	1097	0.18
1200	1196	42.74	1199	0.25	0.9642	1202	0.50
1300	1295	42.84	1295	0	0.9647	1299	0.31
1400	1392	42.95	1411	1.36	0.9651	1421	2.08
1600	1595	43.08	1577	1.13	0.9657	1565	1.88
1700	1691	43.17	1693	0.12	0.9660	1673	1.06
1800	1791	43.24	1800	0.50	0.9664	1773	1.01
1900	1892	43.30	1897	0.26	0.9667	1880	0.63
Average:				0.50			0.95
Variance:				0.51			1.48

^1^ ‘Measured Bitrate’ means the coding bitrate obtained as a result of the encoding process using ffmpeg. ^2^ ‘Interpolated Bitrate’ is a calculated coding bitrate for a video sample of the quality level closest to the presented by the corresponding real video sample and its ‘Measured Bitrate’ (the quality are expressed here by PSNR and SSIM).

**Table 3:** PSNR an SSIM results for the ‘Beauty’ video test samples.

	H.264			H.265			AV1		
Target Bitrate [kbps]	Bitrate ^1^ [kbps]	PSNR [dB]	SSIM	Bitrate [kbps]	PSNR [dB]	SSIM	Bitrate [kbps]	PSNR [dB]	SSIM
500	501	41.042	0.956	505	41.856	0.960	484	42.627	0.964
600	600	41.497	0.958	602	42.259	0.962	559	42.924	0.966
700	699	41.848	0.960	702	42.581	0.964	629	43.156	0.967
800	799	42.123	0.961	801	42.838	0.965	692	43.334	0.967
900	899	42.324	0.962	900	43.041	0.966	754	43.483	0.968
1000	1000	42.482	0.963	1001	43.207	0.967	817	43.615	0.969
1500	1494	43.019	0.965	1502	43.725	0.969	1119	44.045	0.97
2000	1994	43.362	0.967	2007	44.013	0.970	1431	44.328	0.971

^1^ ‘Bitrate’ means the data rate achieved after encoding (measured bitrate).

**Table 4:** PSNR and SSIM results for the ‘ReadyStGo’ video test samples.

	H.264			H.265			AV1		
Target Bitrate [kbps]	Bitrate [kbps]	PSNR [dB]	SSIM	Bitrate [kbps]	PSNR [dB]	SSIM	Bitrate [kbps]	PSNR [dB]	SSIM
500	511	32.812	0.917	526	34.052	0.931	546	36.32	0.953
600	613	33.636	0.928	630	34.825	0.940	653	37.206	0.960
700	716	34.378	0.937	736	35.532	0.947	755	37.921	0.965
800	819	35.033	0.944	841	36.144	0.953	856	38.551	0.969
900	922	35.615	0.950	947	36.704	0.958	956	39.106	0.972
1000	1025	36.146	0.954	1052	37.203	0.961	1058	39.605	0.975
1500	1540	38.188	0.969	1579	39.180	0.973	1532	41.464	0.983
2000	2055	39.710	0.976	2112	40.637	0.980	1887	42.544	0.986

**Table 5:** Bitrate savings for the ‘Beauty’ video footage.

Compared Codecs	ABS_BDR_ [%]		Δ_BDR_ [%]	ABS_DR_ [%]		Δ_DR_ [%]
	based on:			based on:		
	PSNR	SSIM		PSNR	SSIM	
H.264 vs. H.265 *	−35.29	−36.05	0.76	−37.72	−37.48	0.24
H.264 vs. AV1 *	−60.33	−63.02	2.69	−61.33	−62.28	0.95
H.265 vs. AV1 *	−37.57	−40.05	2.48	−39.21	−40.46	1.25
Avg.			1.98			0.81

* Lower bitrate (i.e., better) codec.

**Table 6:** Bitrate savings for the ‘ReadyStGo’ video footage.

Compared Codecs	ABS_BDR_ [%]		Δ_BDR_ [%]	ABS_DR_ [%]		Δ_DR_ [%]
	based on:			based on:		
	PSNR	SSIM		PSNR	SSIM	
H.264 vs. H.265 *	−17.52	−15.22	2.3	−16.48	−14.68	1.8
H.264 vs. AV1 *	−48.20	−44.85	3.35	−48.26	−44.79	3.47
H.265 vs. AV1 *	−38.23	−35.98	2.25	−38.23	−36.20	2.03
Avg.			2.63			2.43

* Lower bitrate (i.e., better) codec.

## Data Availability

The data presented in this study are available on request from the corresponding author.
